# Understanding purchase intention toward upcycled foods through a consumer education lens: an extended theory of planned behavior approach

**DOI:** 10.3389/fnut.2026.1871592

**Published:** 2026-07-24

**Authors:** Liang Shan, Yunhao Zhang, Yuguang Ma, Jing Tian, Youyou Li

**Affiliations:** 1 Network Information Center, Tonghua Normal University, Tonghua, Jilin, China; 2 School of Mathematics and Artificial Intelligence, Tonghua Normal University, Tonghua, Jilin, China; 3 School of Pharmacy and Health Sciences, Tonghua Normal University, Tonghua, Jilin, China; 4 Institute of International Economics and Trade, Liaoning University of International Business and Economics, Dalian, Liaoning, China

**Keywords:** consumer education, environmental awareness, health consciousness, product knowledge, purchase intention, theory of planned behavior, upcycled foods

## Abstract

**Background:**

Upcycled foods offer a promising pathway for reducing food waste, improving resource-use efficiency, and promoting sustainable food consumption by transforming food resources that retain edible or nutritional value into new food products. However, the mechanisms through which consumers form purchase intention toward upcycled foods remain insufficiently understood.

**Methods:**

Drawing on the extended theory of planned behavior, this study developed a research model to examine the effects of health consciousness, environmental awareness, and product knowledge on attitude, as well as the effects of attitude, subjective norm, and perceived behavioral control on purchase intention. The mediating role of attitude was also tested. Data were collected using a three-wave survey design, 406 valid responses were retained for analysis. Exploratory factor analysis, confirmatory factor analysis, structural equation modeling, and bias-corrected bootstrap analysis were used to test the measurement and structural models.

**Results:**

Exploratory factor analysis identified seven factors, consistent with the theoretical constructs proposed in this study. Confirmatory factor analysis indicated satisfactory composite reliability, convergent validity, and discriminant validity for all constructs. The structural equation modeling results showed that health consciousness, environmental awareness, and product knowledge all had significant positive effects on consumers’ attitude toward upcycled foods, with product knowledge showing the strongest effect. Attitude, subjective norm, and perceived behavioral control were also found to positively influence purchase intention. The mediation analysis further showed that attitude significantly mediated the relationships between health consciousness, environmental awareness, product knowledge, and purchase intention.

**Conclusion:**

Health consciousness and environmental awareness provide value-based foundations for consumers’ evaluation of upcycled foods, while product knowledge helps reduce conceptual uncertainty and supports the formation of a favorable attitude. Attitude further links these antecedents to purchase intention, while subjective norm and perceived behavioral control also play important roles in shaping purchase intention. This study extends the application of the theory of planned behavior to the context of emerging sustainable food consumption and provides empirical evidence for the promotion of upcycled foods, consumer education, and sustainable food policy design.

## 1 Introduction

Global food systems are facing interrelated challenges arising from resource depletion, environmental pressure, changing nutritional needs, and shifts in consumption patterns (1). Food security and nutritional provision have traditionally been discussed in relation to insufficient production, unequal distribution, or limited access (2). Yet contemporary food systems also face another problem that is less visible but equally consequential: many food resources that still retain edible value or nutritional potential are not fully used before being lost or wasted across production, processing, distribution, retail, food service, and household consumption. According to the Food Waste Index Report 2024 released by the United Nations Environment Programme, approximately 1.05 billion tons of food waste were generated in the retail, food service, and household sectors worldwide in 2022, equivalent to 19% of the food available to consumers (3). Household food waste accounted for about 631 million tons, representing 60% of total global food waste (4). Food loss and waste are also estimated to contribute 8–10% of global greenhouse gas emissions, indicating that food waste is not only a matter of inefficient resource use but also a climate and sustainability issue (5). These figures suggest that food system transformation cannot rely only on producing more food. It also requires reconsidering how existing food resources are valued, used, communicated, and accepted by consumers.

Upcycled foods provide a practical pathway for addressing this problem. Rather than merely reducing food loss or managing waste at the end of the supply chain, upcycled foods transform food ingredients, by-products, or surplus resources that might otherwise be wasted but still retain edible or nutritional value into new food products through safe, regulated, and innovative processing (6). The central idea is not to repackage low-value materials, but to reidentify and redevelop the value of underused food resources so that they can re-enter the consumption system (7). In this sense, upcycled foods integrate food waste reduction, resource circularity, food innovation, and nutritional value recovery (8). They therefore represent not only a technological or supply-side response to food waste, but also a consumer-facing strategy for promoting sustainable food systems.

However, consumer acceptance of upcycled foods cannot be assumed simply because these products carry sustainability claims. Food consumption is strongly shaped by health concerns, risk sensitivity, trust, and social context (9). When consumers encounter an emerging food category, they often ask whether the product is healthy, safe, trustworthy, consistent with their values, and practically available (10, 11). Upcycled foods involve a particular form of cognitive tension. On the one hand, they communicate positive meanings related to food waste reduction, resource-use efficiency, and sustainable consumption (12). On the other hand, the term “upcycled” may lead some consumers to question ingredient sources, processing methods, product quality, and nutritional value (13). Thus, the key issue is not merely whether consumers are told that upcycled foods are sustainable, but whether they can understand this sustainability claim in a way that supports a favorable product evaluation.

Existing research on sustainable food consumption has generated substantial knowledge about organic foods, green foods, plant-based foods, and functional foods. Yet upcycled foods differ from these more established categories in important ways. Organic and green foods are often associated with relatively clear positive meanings such as naturalness, environmental friendliness, and healthfulness (14). Plant-based and functional foods are commonly evaluated through substitution, health benefits, or functional attributes (15). By contrast, upcycled foods communicate two types of information at the same time: resource reuse and food innovation (16). Resource reuse may encourage consumers to connect their food choices with environmental responsibility and food waste reduction. Food innovation, however, requires consumers to understand why materials that might otherwise be wasted can still become safe, valuable, and acceptable food products. Without such understanding, upcycled foods may be misinterpreted as leftovers, low-quality reprocessed foods, or near-expiry products. This makes consumer education and product knowledge particularly important in this context.

Against this background, health consciousness, environmental awareness, and product knowledge may serve as important antecedents of consumer attitude. Health consciousness reflects the extent to which consumers care about dietary health, nutritional value, and health-related food attributes (17). In food consumption, health concerns often form a basic evaluative filter through which consumers assess unfamiliar products (18). Consumers with strong health consciousness may respond positively to upcycled foods if they understand these products as safe and meaningful ways of reusing food resources that still retain nutritional value. At the same time, health-conscious consumers may also be more cautious if the source or processing logic of the product is unclear. Environmental awareness reflects consumers’ concern for resource waste, environmental consequences, and responsibility for sustainable consumption (19). Since upcycled foods are closely related to reducing food waste and improving resource-use efficiency, environmentally aware consumers may be more likely to recognize their sustainability value. However, environmental concern does not automatically lead to product acceptance when consumers perceive possible trade-offs involving safety, taste, or quality. Product knowledge is therefore especially relevant because it helps consumers understand the concept, sources, processing logic, and consumption meaning of upcycled foods (20). For emerging foods, insufficient knowledge may amplify uncertainty, whereas accurate product knowledge can reduce cognitive barriers and provide a stronger basis for acceptance.

The theory of planned behavior (TPB) provides a useful theoretical foundation for explaining consumers’ purchase intention. According to this theory, behavioral intention is shaped by attitude, subjective norm, and perceived behavioral control (21). Attitude reflects consumers’ overall evaluation of a purchasing behavior, subjective norm captures perceived approval and support from important others or the broader social environment, and perceived behavioral control reflects consumers’ judgment of their ability, resources, and convenience in performing the behavior (22). In the context of upcycled foods, purchase intention may depend not only on whether consumers evaluate this food category positively, but also on whether they perceive social support and whether they believe that such products are accessible and easy to purchase (23). Nevertheless, the classic TPB gives relatively limited attention to how attitude is formed. This issue is important for upcycled foods because attitude formation is closely tied to how consumers interpret health value, environmental value, and product information. Therefore, this study extends TPB by introducing health consciousness, environmental awareness, and product knowledge as antecedents of attitude.

Accordingly, this study addresses the following research questions:

RQ1. How do health consciousness, environmental awareness, and product knowledge shape consumers’ attitudes toward upcycled foods?

RQ2. How do attitude, subjective norm, and perceived behavioral control influence consumers’ purchase intention toward upcycled foods?

RQ3. Does attitude mediate the relationships between health consciousness, environmental awareness, product knowledge, and purchase intention toward upcycled foods?

This study contributes to the literature in several ways. It extends sustainable food consumption research to upcycled foods, an emerging category that combines resource reuse, nutritional value recovery, and consumer cognitive uncertainty. It also advances the application of the theory of planned behavior by examining how attitudes toward upcycled foods are formed, rather than focusing only on whether attitudes predict purchase intention. By incorporating health consciousness, environmental awareness, and product knowledge as antecedents of attitude, the model offers a clearer explanation of why consumers may evaluate upcycled foods positively or negatively. The mediation analysis further identifies a “cognition–evaluation–intention” pathway through which these antecedents become linked to purchase intention. The findings also have practical and policy relevance. They indicate that promoting upcycled foods requires more than general sustainability claims. Consumers need clear and credible information about ingredient sources, nutritional value, processing safety, and environmental benefits. These insights can inform consumer education, market communication, labeling and information disclosure practices, food waste reduction campaigns, and policies designed to support more sustainable food systems.

## 2 Literature review and hypotheses

### 2.1 Extended theory of planned behavior

The theory of planned behavior (TPB) provides a well-established framework for explaining how individuals form behavioral intentions. According to TPB, behavioral intention is primarily shaped by attitude, subjective norm, and perceived behavioral control (21). Attitude refers to an individual’s overall evaluation of performing a given behavior; subjective norm captures the perceived support or expectations of important others and the broader social environment; and perceived behavioral control reflects an individual’s assessment of the ability, resources, and conditions required to perform the behavior (22). Rather than explaining consumer behavior solely through product attributes or external stimuli, TPB emphasizes the joint role of psychological evaluation, social influence, and perceived behavioral feasibility. For this reason, it has been widely applied to studies of food purchasing, healthy eating, and sustainable consumption (24, 25).

Consumer acceptance of upcycled foods has distinctive contextual features. Consumers are not evaluating a fully familiar conventional food product, but an emerging category that combines resource reuse, food innovation, and sustainable consumption meanings (26). Purchase intention toward upcycled foods may be encouraged by their positive environmental value, but it may also be constrained by conceptual unfamiliarity, insufficient information, or concerns about ingredient sources (27). In this context, attitude, subjective norm, and perceived behavioral control can explain whether consumers evaluate the purchase of upcycled foods favorably, whether they perceive support from their social environment, and whether they believe they have the conditions and ability to purchase such products (28). TPB therefore provides a useful theoretical foundation for examining purchase intention toward upcycled foods.

However, the classic TPB primarily focuses on the direct effects of attitude, subjective norm, and perceived behavioral control on behavioral intention, while paying relatively limited attention to how attitude itself is formed. This issue is particularly important in the context of upcycled foods. Whether consumers develop a favorable attitude depends largely on how they understand the health meaning, environmental value, and product attributes of this food category (29, 30). Consumers with higher health consciousness may pay closer attention to whether upcycled foods have nutritional value and health-related attributes (31). Consumers with higher environmental awareness may be more likely to recognize their role in reducing food waste and improving resource-use efficiency (32). Consumers with greater product knowledge may better understand the concept, sources, and processing logic of upcycled foods, thereby reducing uncertainty caused by unfamiliarity (33).

Based on this reasoning, the present study extends TPB by introducing health consciousness, environmental awareness, and product knowledge as antecedents of attitude. This extension is not merely the addition of external variables; rather, it opens up the antecedent process through which attitude is formed. It explains how consumers develop an overall evaluation of upcycled foods based on health concerns, environmental value orientation, and product understanding, and how this evaluation is further linked to purchase intention. By integrating the classic TPB pathways with the contextual characteristics of upcycled foods, this study provides a more targeted explanatory framework for understanding emerging sustainable food consumption behavior.

### 2.2 Health consciousness and attitude

Health consciousness refers to the extent to which consumers are concerned about their health status, dietary quality, and the health consequences of food choices (34). In food consumption contexts, health consciousness functions as more than a general preference for healthy living. It shapes how consumers attend to food-related information, what product attributes they consider important, and how they evaluate unfamiliar food categories (35). Consumers with higher health consciousness tend to pay closer attention to nutritional composition, processing methods, functional attributes, and potential long-term health effects (36). Food choice, for these consumers, is often viewed as part of a broader effort to maintain or improve personal health rather than as a purely routine consumption decision.

However, the relationship between health consciousness and attitude toward emerging foods is not necessarily straightforward. Health-conscious consumers may be more receptive to products that offer clear nutritional value, but they may also be more cautious when ingredient sources, processing logic, or safety assurance are unclear (37). In this sense, health consciousness can increase both acceptance and scrutiny. This tension is particularly relevant to upcycled foods. Although upcycled foods are often promoted through the language of food waste reduction and resource circularity, consumers may still ask whether such products are safe, nutritious, and suitable for regular consumption (12, 13). If the concept of upcycling is poorly understood, health-conscious consumers may hesitate rather than respond positively.

The positive role of health consciousness is therefore likely to depend on how consumers interpret the meaning of upcycled foods. Many upcycled foods are derived from food ingredients, by-products, or surplus resources that still retain edible value or nutritional potential and are reintroduced into the consumption system through regulated processing and product innovation (38). When consumers understand upcycled foods as a safe and meaningful way to recover nutritional value from underused food resources, the attribute of “upcycling” may acquire a more favorable health-related meaning. Under this condition, health consciousness can encourage consumers to evaluate upcycled foods not merely as sustainable products, but also as food choices that may be compatible with health-oriented consumption. Thus, consumers who are more concerned about dietary health are more likely to form a favorable attitude toward upcycled foods when they perceive these products as nutritionally reasonable and safely processed (39). Accordingly, this study proposes the following hypothesis:

*H1*: Health consciousness positively influences consumers’ attitude toward upcycled foods.

### 2.3 Environmental awareness and attitude

Environmental awareness reflects consumers’ concern about environmental issues, resource consumption, and the ecological consequences of their own consumption behavior (40). In food consumption contexts, it shapes how consumers interpret the value of food products beyond immediate attributes such as price, taste, and convenience (41). Consumers with higher environmental awareness are more likely to consider the environmental burdens generated during food production, processing, distribution, and consumption. They may also be more sensitive to whether a product contributes to resource conservation, waste reduction, or lower environmental impact (26). In this respect, environmental awareness can provide a value-based foundation for evaluating sustainable food products.

However, the influence of environmental awareness on food evaluation should not be understood as automatic. Prior research on sustainable consumption has shown that consumers may express concern for the environment while still hesitating to purchase sustainable products when they perceive trade-offs involving food safety, taste, price, convenience, or product quality (19, 29). This attitude–behavior tension is particularly relevant to upcycled foods. Although these products are closely associated with food waste reduction and resource circularity, consumers may still question whether the environmental value of upcycling is sufficient to offset uncertainty about ingredient sources or processing methods (6, 7). Thus, environmental awareness may encourage consumers to pay attention to upcycled foods, but a favorable attitude is more likely to emerge when the environmental meaning of these products is perceived as concrete, credible, and compatible with acceptable food quality.

Upcycled foods are nevertheless strongly aligned with the concerns of environmentally aware consumers. Their core value lies in bringing food resources that might otherwise be wasted back into the consumption system, thereby extending the value chain of food resources and reducing unnecessary resource loss (42). For consumers who are concerned about food waste and resource consumption, purchasing upcycled foods may be interpreted not merely as trying a new product, but as a practical form of participation in sustainable food systems. Compared with abstract environmental beliefs, upcycled foods provide a more direct consumption context in which consumers can connect everyday food choices with resource conservation and environmental responsibility (23). Therefore, when consumers recognize the role of upcycled foods in reducing food waste and improving resource-use efficiency, environmental awareness is likely to support a more favorable attitude toward this food category (43). Accordingly, this study proposes the following hypothesis:

*H2*: Environmental awareness positively influences consumers’ attitude toward upcycled foods.

### 2.4 Product knowledge and attitude

Product knowledge refers to consumers’ understanding of a product category, including its concept, attributes, sources, uses, and consumption value (44). For emerging foods, product knowledge is especially important because consumers often have limited direct experience and therefore rely more heavily on available information when forming attitudes (10). Upcycled foods remain relatively unfamiliar to many consumers, and the term “upcycled” itself may create conceptual ambiguity. Without sufficient understanding of ingredient sources, processing logic, and the value of food resource reuse, consumers may misinterpret upcycled foods as leftover food disposal, low-quality reprocessed food, or near-expiry food (45). Such misunderstanding can easily weaken product evaluation, even when the product is intended to deliver sustainability benefits.

The role of product knowledge, however, is not simply that more information always leads to more favorable attitudes. Knowledge may reduce uncertainty, but it may also make consumers more attentive to processing details, ingredient origins, and possible quality differences (20). For a food category that involves the reuse of underutilized resources, consumers may become more critical if the information they receive is vague, inconsistent, or difficult to verify (44). Therefore, the key issue is not merely whether consumers have heard of upcycled foods, but whether they have accurate knowledge that helps them distinguish upcycled foods from waste disposal, leftovers, or low-quality reprocessing. In this sense, product knowledge has a corrective function: it can reshape the meaning of “upcycling” from a potentially ambiguous label into an understandable form of food resource redevelopment (45).

Accurate product knowledge also helps consumers build clearer cognitive links among food resource utilization, nutritional value, and sustainability (46). Consumers who understand upcycled foods are more likely to recognize that these products are not simply repackaged waste materials, but new consumer products created by transforming food resources that still retain edible value or nutritional potential through safe processing and product innovation (33). This understanding can strengthen judgments about the reasonableness, acceptability, and consumption value of upcycled foods, while reducing concerns about ingredient sources and product quality. Product knowledge therefore functions as an important cognitive foundation for attitude formation, particularly in a market context where consumer education remains necessary (47). When consumers better understand the concept, sources, processing methods, and sustainability value of upcycled foods, they are more likely to evaluate this food category as reasonable, meaningful, and acceptable (48). Accordingly, this study proposes the following hypothesis:

*H3*: Product knowledge positively influences consumers’ attitude toward upcycled foods.

### 2.5 Attitude, subjective norm, perceived behavioral control, and purchase intention

Purchase intention refers to consumers’ perceived likelihood of buying a product in the future and is a key outcome variable in research on market acceptance of emerging foods (49). In the context of upcycled foods, purchase intention is shaped not only by consumers’ evaluation of the value and meaning of this food category, but also by the social environment and perceived feasibility of purchase (27). According to the theory of planned behavior, attitude, subjective norm, and perceived behavioral control jointly constitute the main psychological foundations of behavioral intention. In this study, these three factors correspond to consumers’ evaluation of purchasing upcycled foods, perceived influence from social relationships and the broader social environment, and judgments about the conditions and control required to perform the purchase behavior (28).

Attitude represents consumers’ overall evaluation of purchasing upcycled foods. When consumers perceive the purchase of upcycled foods as valuable, meaningful, and consistent with their consumption needs, they are more likely to develop stronger purchase intention (26). Because upcycled foods are still an emerging food category, consumers often need to evaluate their health value, environmental meaning, and product legitimacy before forming purchase intention. A favorable attitude indicates that consumers have, to some extent, accepted the consumption value of upcycled foods and reduced the psychological distance associated with this new food category (12). At the same time, attitude toward an emerging food category may remain fragile if consumers still have unresolved concerns about safety, taste, or product quality. Thus, a positive attitude is important because it suggests that consumers have moved beyond initial unfamiliarity and are more willing to consider upcycled foods as a reasonable purchase option. Accordingly, this study proposes the following hypothesis:

*H4*: Attitude positively influences consumers’ purchase intention toward upcycled foods.

Subjective norm refers to consumers’ perception of support and approval from important others or the broader social environment regarding their purchase behavior. Although food consumption involves personal choice, it is not fully independent of social influence. Family members, friends, peers, expert recommendations, media information, and social advocacy may all shape consumers’ acceptance of emerging foods (50). For upcycled foods, consumers’ direct experience is often limited, making external social cues particularly relevant for reducing uncertainty and strengthening the perceived legitimacy of purchase (23). However, social influence may not always translate into purchase intention if consumers perceive the product as unfamiliar, risky, or inconsistent with their own food preferences. Its effect is more likely to emerge when social approval helps reduce hesitation and makes the purchase of upcycled foods appear acceptable and meaningful. When consumers perceive that important others support their purchase of upcycled foods, or when they believe that the surrounding social environment holds a positive view of sustainable food consumption, their psychological resistance to purchasing such products may decline and purchase intention may become stronger (51). Accordingly, this study proposes the following hypothesis:

*H5*: Subjective norm positively influences consumers’ purchase intention toward upcycled foods.

Perceived behavioral control reflects consumers’ subjective judgment of the ability, resources, and opportunities required to complete a purchase behavior. Even when consumers hold a favorable attitude toward upcycled foods and perceive social support, purchase intention may still be constrained if they believe that such products are difficult to access, that information is insufficient, that prices are unacceptable, or that purchase channels are limited (12). Conversely, when consumers believe that they have the necessary information, financial capacity, and channel access, and that the purchase process is relatively convenient, the behavior is more likely to be perceived as feasible. For upcycled foods, which have not yet become fully mainstream, perceived behavioral control reflects not only individual capability but also the role of market availability, information transparency, and purchase convenience in shaping consumer decision-making (28). This factor is particularly important because consumer acceptance of upcycled foods may remain at the level of positive evaluation if products are not visible, accessible, or easy to identify in actual purchase settings. Accordingly, this study proposes the following hypothesis:

*H6*: Perceived behavioral control positively influences consumers’ purchase intention toward upcycled foods.

### 2.6 The mediating role of attitude

Health consciousness, environmental awareness, and product knowledge provide different cognitive foundations for consumers’ understanding of upcycled foods, corresponding, respectively, to health value, environmental responsibility, and product understanding. However, these factors do not necessarily translate into purchase intention in a direct or automatic manner. In the case of upcycled foods, consumers are not merely facing a new product with sustainable attributes; they are evaluating a food category that requires interpretation, comparison, and reassessment (33, 45). Health-conscious consumers may pay closer attention to nutritional value and processing safety (52), but such attention may also make them more cautious if the product information is unclear. Environmentally aware consumers may be sensitive to the role of upcycled foods in reducing food waste and improving resource-use efficiency (53), but environmental concern may be weakened if consumers perceive possible trade-offs involving safety, taste, or quality. Consumers with greater product knowledge may better understand the concept, sources, and consumption value of upcycled foods (54), but this understanding still needs to be integrated into an overall product evaluation before it can support purchase intention. Therefore, the relationship between these antecedents and purchase intention requires further explanation through attitude.

Attitude plays a psychological conversion role in this process by integrating value concerns and product cognition into an overall evaluation of upcycled foods. Health consciousness may encourage consumers to evaluate upcycled foods from the perspectives of nutritional utilization and health-oriented consumption; when consumers perceive such foods as consistent with health-related dietary needs, their purchase intention is more likely to be strengthened (55). Environmental awareness may increase consumers’ sensitivity to the value of resource circularity and food waste reduction; when this environmental concern is translated into a favorable evaluation of upcycled foods, consumers are more likely to consider them as future food choices (48). Product knowledge may reduce conceptual unfamiliarity and cognitive uncertainty, helping consumers avoid interpreting upcycled foods simply as leftovers or low-quality reprocessed foods and instead recognize them as a reasonable and valuable emerging food category (56). In this sense, attitude serves as a plausible pathway through which health-related concerns, environmental values, and product understanding become more closely linked to behavioral intention. Based on this reasoning, the following hypotheses are proposed:

*H7a*: Attitude mediates the relationship between health consciousness and consumers’ purchase intention toward upcycled foods.

*H7b*: Attitude mediates the relationship between environmental awareness and consumers’ purchase intention toward upcycled foods.

*H7c*: Attitude mediates the relationship between product knowledge and consumers’ purchase intention toward upcycled foods.

**Figure 1:**
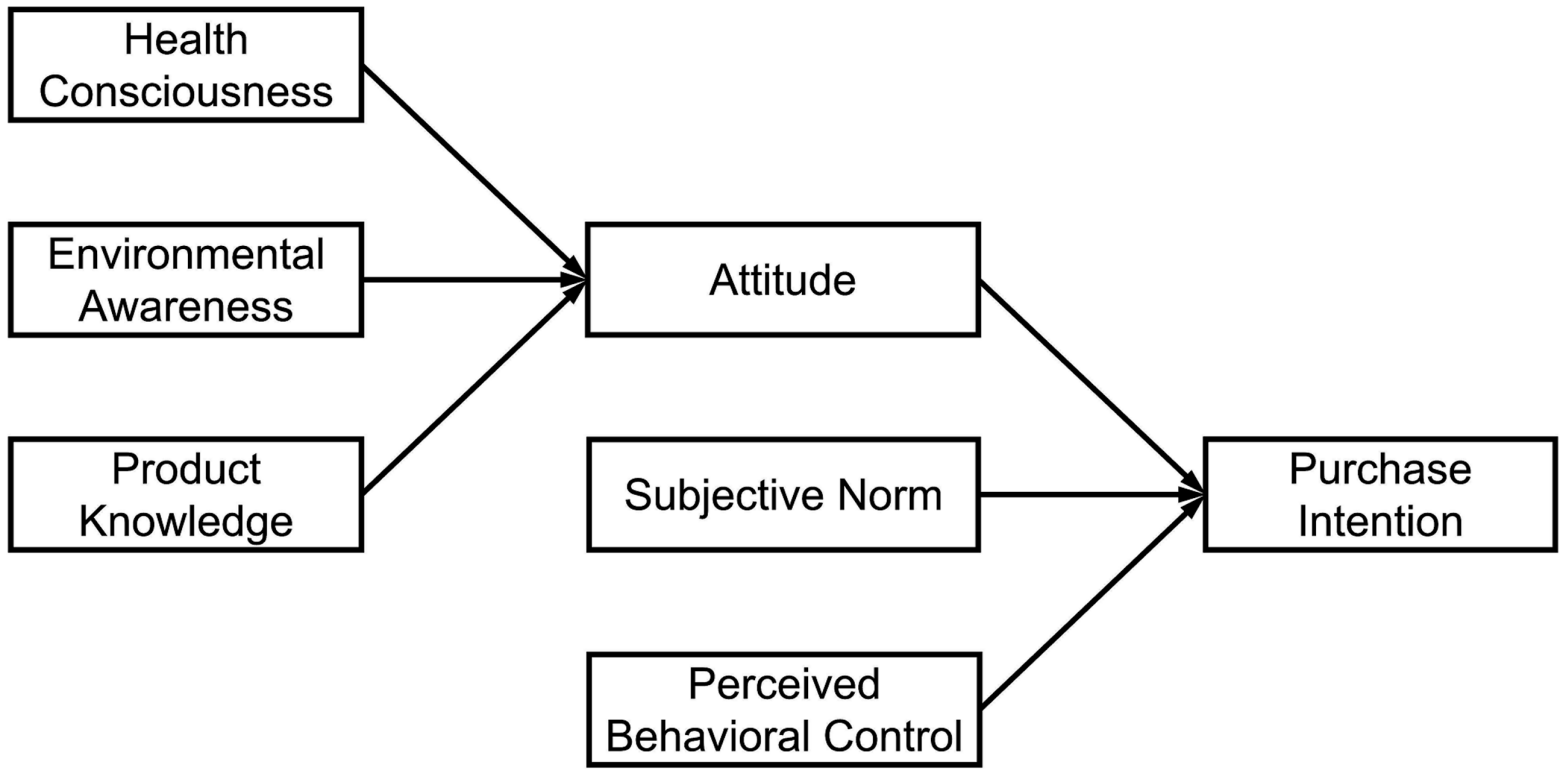
Research model.

Based on the above theoretical arguments and hypotheses, this study develops a conceptual model of consumers’ purchase intention toward upcycled foods. Health consciousness, environmental awareness, and product knowledge are specified as antecedents of attitude, while attitude, subjective norm, and perceived behavioral control are specified as direct predictors of purchase intention. Attitude further links health consciousness, environmental awareness, and product knowledge with purchase intention, reflecting the psychological evaluation process through which consumers move from value cognition and product understanding to behavioral intention. The proposed research model is presented in Figure 1.

## 3 Methodology

### 3.1 Research design

This study used a structured questionnaire to collect data, focusing on consumers’ cognition, evaluation, and purchase intention toward upcycled foods. At the beginning of the questionnaire, respondents were informed about the research purpose, response requirements, and data use. The questionnaire clearly stated that the survey was conducted solely for academic purposes, involved no commercial use, and did not collect personally identifiable information. Participation was voluntary and based on informed consent, and respondents could withdraw from the survey at any time. To reduce privacy concerns and potential social desirability bias, all responses were collected anonymously, and the results were reported only in aggregate form.

All latent constructs were measured using items adapted from established scales and modified to fit the context of upcycled food consumption. Health consciousness measured consumers’ concern about the health consequences of their daily diet, nutritional value, and health-related food attributes, with items adapted from prior studies on health consciousness (57, 58). Environmental awareness captured consumers’ concern about the environmental impact of food consumption, resource waste, and food waste, with items adapted from studies on environmental awareness and sustainable food consumption (59, 60). Product knowledge measured consumers’ understanding of the concept, sources, and consumption value of upcycled foods, with items adapted from research on consumer product knowledge and food knowledge (61, 62).

Attitude, subjective norm, and perceived behavioral control were measured based on the theory of planned behavior and its applications in food consumption research (63, 64). Attitude reflected consumers’ overall evaluation of purchasing upcycled foods. Subjective norm captured consumers’ perceived support and approval from important others or people around them regarding the purchase of upcycled foods. Perceived behavioral control reflected consumers’ perceived ability, convenience, information access, and resource conditions related to purchasing upcycled foods. Purchase intention was treated as the outcome variable and measured consumers’ likelihood of purchasing, considering, and trying upcycled foods in the future, with items adapted from studies on sustainable food consumption and purchase intention (60, 65, 66).

Except for demographic information, all core variables were measured using a five-point Likert scale ranging from 1 = strongly disagree to 5 = strongly agree. Demographic information included gender, age, educational level, and monthly income, which were used to describe the sample profile. Before the formal survey, the questionnaire items were reviewed and revised for semantic clarity and contextual suitability to improve item comprehensibility and measurement accuracy. The full list of measurement items is presented in Appendix A.

### 3.2 Sampling and data collection

Primary data were collected through an online questionnaire using a non-probability purposive sampling technique. This approach was adopted because the study required respondents to be adult consumers with experience in daily food purchasing or consumption decision-making. Participants were recruited through an online survey platform and were required to be at least 18 years old. Comprehension confirmation items and attention-check questions were included to ensure that respondents understood the research context and completed the questionnaire carefully. Before participation, respondents were informed of the academic purpose of the study, the voluntary nature of participation, and the anonymous treatment of their responses.

Data were collected in three waves, with an interval of approximately 2 weeks between each wave. In the first wave, health consciousness, environmental awareness, product knowledge, and demographic information were measured, and 494 responses were obtained. In the second wave, attitude, subjective norm, and perceived behavioral control were measured, resulting in 461 responses. Purchase intention was measured in the third wave, in which 447 responses were collected. Anonymous identification codes were used to match responses across the three waves. After matching the data and removing responses that failed the quality-screening criteria, 406 valid cases were retained for further analysis.

The final sample size was considered adequate for structural equation modeling. The questionnaire contained 28 measurement items excluding demographic questions, and the sample of 406 respondents exceeded the commonly recommended minimum of 10 observations per item. The sample also included consumers from different gender, age, educational, and income groups. Of the respondents, 46.06% were male and 53.94% were female. The largest age group was 30–40 years (30.30%), while 35.96% held a bachelor’s degree. Regarding monthly income, the largest proportion of respondents reported an income between RMB 9,001 and RMB 12,000 (28.08%). The complete demographic profile of the respondents is presented in Table 1.

**Table 1:** Demographics profile of the respondents.

Variable	Category	Respondents	
		Frequency (*n* = 406)	Percentage
Gender	Male	187	46.06
	Female	219	53.94
Age (in years)	18–30	101	24.88
	30–40	123	30.30
	40–50	88	21.67
	50–60	65	16.01
	Above 60	29	7.14
Educational qualification	High school or below	45	11.08
	Associate Degree	117	28.82
	Bachelor’s Degree	146	35.96
	Master’s Degree and Above	98	24.14
Monthly Income (in rmb)	Less than 3,000	34	8.37
	3,001–6,000	76	18.72
	6,001–9,000	89	21.92
	9,001–12,000	114	28.08
	More than 12,000	93	22.91

### 3.3 Data analysis

The data collected through the questionnaires were analyzed using SPSS and AMOS software. Exploratory factor analysis was first conducted to examine the underlying factor structure of the measurement items, and confirmatory factor analysis was subsequently performed to confirm the proposed seven-factor structure. Convergent validity indicates whether the measurement items are adequately correlated with their respective constructs, whereas discriminant validity assesses whether the constructs are empirically distinct from one another. Cronbach’s alpha and composite reliability were used to assess internal consistency, while standardized factor loadings and average variance extracted were used to evaluate convergent validity. Structural equation modeling was employed to test the hypothesized relationships among the constructs. The bias-corrected bootstrapping method with 5,000 resamples was used to examine the mediating role of attitude, and an indirect effect was considered significant when the corresponding 95% bootstrap confidence interval did not include zero.

## 4 Results

### 4.1 Exploratory factor analysis

Exploratory factor analysis was conducted to examine the underlying structure of the measurement items. The results showed a KMO value of 0.951, indicating that the data were highly suitable for factor analysis. Bartlett’s test of sphericity was significant (*p* < 0.001), suggesting sufficient correlations among the items for factor extraction.

Principal components were extracted using the eigenvalue-greater-than-one criterion, and varimax rotation was applied to improve factor interpretability. Seven factors were extracted, corresponding to the seven theoretical constructs in this study: health consciousness, environmental awareness, product knowledge, attitude, subjective norm, perceived behavioral control, and purchase intention. The rotated variance explained by the seven factors was 11.178%, 10.703%, 10.631%, 10.601%, 10.423%, 10.237%, and 10.217%, respectively, with a cumulative explained variance of 73.991%. These results indicate that the extracted factors captured a substantial proportion of the information contained in the measurement items. In addition, the communalities of all items were above 0.40, showing that the items were adequately explained by the common factors. Overall, the exploratory factor analysis supported the seven-factor structure of the measurement scale.

### 4.2 Confirmatory factor analysis

Confirmatory factor analysis was further conducted to assess the reliability, convergent validity, and discriminant validity of the measurement model. Table 2 presents the standardized factor loadings, average variance extracted (AVE), Cronbach’s *α*, and composite reliability (CR) for each construct. The standardized factor loadings ranged from 0.727 to 0.881, all exceeding the recommended threshold of 0.70, indicating that the items adequately represented their respective latent constructs.

**Table 2:** Reliability and convergent validity statistics.

Constructs	Items	Factor loadings	Cronbach’s α	CR	AVE
Health consciousness	HC1	0.840	0.894	0.896	0.683
	HC2	0.881			
	HC3	0.811			
	HC4	0.768			
Environmental awareness	EA1	0.831	0.884	0.884	0.656
	EA2	0.806			
	EA3	0.784			
	EA4	0.817			
Product knowledge	PK1	0.794	0849	0.849	0.585
	PK2	0.765			
	PK3	0.729			
	PK4	0.770			
Attitude	ATT1	0.838	0.866	0.868	0.622
	ATT2	0.795			
	ATT3	0.790			
	ATT4	0.727			
Subjective norm	SN1	0.827	0.875	0.877	0.64
	SN2	0.826			
	SN3	0.800			
	SN4	0.744			
Perceived behavioral control	PBC1	0.838	0.888	0.890	0.669
	PBC2	0.865			
	PBC3	0.804			
	PBC4	0.761			
Purchase intention	PI1	0.852	0.887	0.888	0.665
	PI2	0.814			
	PI3	0.807			
	PI4	0.787			

CR, composite reliability; AVE, average variance extracted.

The results also supported the reliability and convergent validity of the measurement model. Cronbach’s α values ranged from 0.849 to 0.894, and the CR values ranged from 0.849 to 0.896, all exceeding the recommended threshold of 0.70. The AVE values ranged from 0.585 to 0.683, all above the threshold of 0.50. These results indicate satisfactory internal consistency and convergent validity across all constructs, suggesting that the measurement model captured the proposed theoretical constructs reliably.

Table 3 reports the Pearson correlation coefficients among the constructs and the square roots of AVE. Discriminant validity was assessed using the Fornell–Larcker criterion, which requires the square root of AVE for each construct to be greater than its correlations with other constructs. The square roots of AVE ranged from 0.765 to 0.826 and were higher than the corresponding inter-construct correlations. This result indicates that the constructs maintained sufficient empirical distinctiveness.

**Table 3:** Discriminant validity statistics.

Constructs	HC	EA	PK	ATT	SN	PBC	PI
HC	0.826						
EA	0.579	0.810					
PK	0.563	0.630	0.765				
ATT	0.551	0.542	0.553	0.789			
SN	0.516	0.552	0.499	0.558	0.800		
PBC	0.461	0.573	0.551	0.588	0.618	0.818	
PI	0.500	0.526	0.520	0.625	0.609	0.637	0.815

HC, health consciousness; EA, environmental awareness; PK, product knowledge; ATT, attitude; SN, subjective norm; PBC, perceived behavioral control; PI, purchase intention.

### 4.3 Structural equation modeling

Structural equation modeling was used to test the hypothesized relationships among the constructs. As shown in the Table 4, the model fit indices indicated that the structural model fitted the data well: *χ*^2^/df = 1.635, which was below the commonly recommended threshold of 3; GFI = 0.916, CFI = 0.971, NFI = 0.930, and NNFI = 0.968, all exceeding 0.90; and RMSEA = 0.040 and SRMR = 0.048, both below 0.08. These results suggest that the structural model was appropriate for examining the proposed theoretical relationships.

**Table 4:** Goodness of fit statistics.

Model-fit statistics	χ^2^/df	GFI	CFI	NFI	NNFI	RMSEA	SRMR
Values	1.635	0.916	0.971	0.930	0.968	0.040	0.048

*χ*^2^/df, chi-square divided by degrees of freedom; GFI, goodness-of-fit index; CFI, comparative fit index; NFI, normed fit index; NNFI, non-normed fit index; RMSEA, root mean square error of approximation; SRMR, standardized root mean square residual.

Table 5 reports the path coefficients and significance levels for the structural model. Health consciousness had a significant positive effect on attitude (*β* = 0.273, *p* < 0.001), indicating that consumers who paid greater attention to dietary health and health-related food attributes were more likely to form favorable evaluations of upcycled foods. Thus, H1 was supported. Environmental awareness also had a significant positive effect on attitude (*β* = 0.195, *p* = 0.010), suggesting that consumers’ concern about resource waste and environmental consequences enhanced their acceptance of upcycled foods. Therefore, H2 was supported. Product knowledge showed a significant positive effect on attitude (*β* = 0.348, *p* < 0.001) and had the largest standardized coefficient among the three antecedents of attitude. This result indicates that consumers’ understanding of the concept, sources, and value of upcycled foods is an important cognitive basis for developing a favorable attitude. Thus, H3 was supported.

**Table 5:** Regression coefficients of the structural model.

Hypothesis	Path	SE	*z* value	*p* value	β
H1	HC → ATT	0.068	4.310	0.000	0.273
H2	EA → ATT	0.072	2.562	0.010	0.195
H3	PK → ATT	0.078	4.421	0.000	0.348
H4	ATT → PI	0.049	7.158	0.000	0.361
H5	SN → PI	0.057	4.163	0.000	0.257
H6	PBC → PI	0.055	5.239	0.000	0.325

HC, health consciousness; EA, environmental awareness; PK, product knowledge; ATT, attitude; SN, subjective norm; PBC, perceived behavioral control; PI, purchase intention; SE, standard error; β, standardized path coefficient.

Attitude had a significant positive effect on purchase intention (*β* = 0.361, *p* < 0.001), showing that consumers with more favorable overall evaluations of upcycled foods were more likely to express future purchase intention. H4 was therefore supported. Subjective norm also positively influenced purchase intention (*β* = 0.257, *p* < 0.001), indicating that approval and support from important others or the surrounding social environment strengthened consumers’ willingness to purchase upcycled foods. Thus, H5 was supported. Perceived behavioral control exerted a significant positive effect on purchase intention (*β* = 0.325, *p* < 0.001), suggesting that consumers were more likely to develop purchase intention when they perceived that they had the necessary information, resources, and ability to purchase upcycled foods. Accordingly, H6 was supported.

### 4.4 Mediation analysis

This study used the bias-corrected bootstrap method to examine the mediating role of attitude in the relationships between health consciousness, environmental awareness, product knowledge, and purchase intention. The number of bootstrap resamples was set to 5,000. This method directly estimates confidence intervals for indirect effects and is therefore suitable for assessing the significance of mediation effects. A mediation effect is considered significant when the 95% bootstrap confidence interval does not include zero. Table 6 presents the results of the mediation analysis.

**Table 6:** Results of the mediation analysis.

Hypothesis	Path	Indirect effect	Boot SE	z value	95% Boot CI
H7a	HC → ATT → PI	0.117	0.030	3.921	0.062 ~ 0.179
H7b	EA → ATT → PI	0.081	0.027	2.952	0.037 ~ 0.145
H7c	PK → ATT → PI	0.106	0.026	4.018	0.058 ~ 0.161

HC, health consciousness; EA, environmental awareness; PK, product knowledge; ATT, attitude; PI, purchase intention; Boot SE, bootstrap standard error; Boot CI, bootstrap confidence interval.

Health consciousness had a significant indirect effect on purchase intention through attitude, with an indirect effect value of 0.117 and a 95% bootstrap confidence interval of [0.062, 0.179]. Because the confidence interval did not include zero, H7a was supported. This result indicates that consumers’ concern about dietary health and health-related food attributes can strengthen purchase intention by improving their attitude toward upcycled foods.

Environmental awareness also had a significant indirect effect on purchase intention through attitude, with an indirect effect value of 0.081 and a 95% bootstrap confidence interval of [0.037, 0.145]. The confidence interval did not include zero, supporting H7b. This finding suggests that consumers’ concern about food waste, resource use, and environmental consequences can promote a favorable attitude toward upcycled foods, which in turn enhances purchase intention.

Product knowledge exerted a significant indirect effect on purchase intention through attitude, with an indirect effect value of 0.106 and a 95% bootstrap confidence interval of [0.058, 0.161]. Since the confidence interval did not include zero, H7c was supported. This result shows that consumers who better understand the concept, sources, and consumption value of upcycled foods are more likely to develop a favorable attitude, which further contributes to purchase intention.

## 5 Discussion

Drawing on the extended theory of planned behavior, this study examined how health consciousness, environmental awareness, and product knowledge shape consumers’ attitudes toward upcycled foods, and how attitude, subjective norm, and perceived behavioral control influence purchase intention. The empirical results supported the proposed hypotheses, indicating that consumers’ purchase intention toward upcycled foods is not driven by a single factor. Instead, it is shaped by health-related value judgments, environmental responsibility, product understanding, attitudinal evaluation, social influence, and perceived behavioral feasibility. For an emerging food category such as upcycled foods, consumer acceptance involves not only recognition of its sustainability value but also broader judgments about its food attributes, consumption legitimacy, and purchase conditions.

Health consciousness had a significant positive effect on consumers’ attitude toward upcycled foods. This finding suggests that consumers who are more concerned about the health consequences of their daily diet are more likely to form favorable evaluations of upcycled foods. Although upcycled foods are often discussed in relation to food waste reduction and resource circularity, food consumption itself is strongly health-oriented (67). When evaluating emerging food products, consumers usually consider not only environmental attributes but also whether the product fits their health-related dietary needs. For consumers with higher health consciousness, if upcycled foods are understood as a regulated use of food resources that still retain edible value or nutritional potential, the attribute of “upcycling” may acquire positive nutritional and health-related meanings (31). This result indicates that consumer acceptance of upcycled foods depends not only on environmental value but also on whether these products can be incorporated into consumers’ health-oriented food choice framework.

The positive effect of environmental awareness on attitude was also confirmed. Consumers who are more concerned about resource waste and environmental consequences are more likely to recognize the value of upcycled foods (39). The core meaning of upcycled foods lies in bringing food resources that might otherwise be wasted back into the consumption system, thereby extending the value chain of food resources and reducing unnecessary resource loss. Consumers with stronger environmental awareness are more likely to interpret the purchase of upcycled foods as a concrete sustainable consumption behavior rather than merely as trying a new product (23). This finding suggests that, in the context of upcycled foods, environmental awareness helps consumers connect personal food choices with food waste reduction, resource conservation, and the development of sustainable food systems, thereby fostering a more favorable product attitude.

Product knowledge showed the strongest effect on attitude, highlighting the distinctive contextual characteristics of consumer acceptance of upcycled foods. Compared with more familiar categories such as organic foods and green foods, upcycled foods are still more susceptible to conceptual unfamiliarity and cognitive confusion (45). If consumers do not understand their ingredient sources, processing logic, and consumption value, they may misinterpret upcycled foods as leftovers, low-quality reprocessed foods, or near-expiry products. Product knowledge helps consumers develop a more accurate cognitive framework and understand that upcycled foods are not simply the processing of low-value materials, but the redevelopment of food resources that still retain edible value or nutritional potential (68). This result indicates that consumers’ favorable attitudes toward upcycled foods depend substantially on whether they truly understand the conceptual boundaries and product value of this food category.

Attitude, subjective norm, and perceived behavioral control all had significant positive effects on purchase intention, supporting the applicability of the theory of planned behavior in the context of upcycled food consumption. The significant effect of attitude indicates that consumers are more likely to develop purchase intention when they form favorable evaluations of upcycled foods (56). For emerging food categories, a positive attitude represents more than general preference (69); it also indicates that consumers have to some extent accepted the legitimacy, value, and consumption meaning of the product category (70). The significant role of subjective norm suggests that purchasing upcycled foods is not determined solely by independent individual judgment. Because consumers often have limited direct experience with this food category, support from family members, friends, experts, media, or a broader social climate favoring sustainable consumption can reduce psychological resistance to trial and strengthen the perceived social legitimacy of purchase (71). The significant effect of perceived behavioral control further shows that purchase intention also depends on whether consumers perceive the behavior as feasible. If products are difficult to access, information is insufficient, purchase channels are limited, or decision costs are high, purchase intention may be constrained even when consumers hold favorable attitudes (72).

The mediation results further reveal the psychological conversion role of attitude between the antecedent variables and purchase intention. Within the theoretical model proposed in this study, health consciousness, environmental awareness, and product knowledge were all indirectly associated with purchase intention through attitude. This suggests that consumers’ health concerns, environmental value orientation, and product cognition can become linked to purchase intention through attitudinal evaluation (73). The indirect effect of health consciousness through attitude indicates that concern for healthy eating becomes associated with purchase intention when consumers develop a favorable evaluation of upcycled foods (67). The indirect effect of environmental awareness through attitude suggests that environmental concern can enter the consumer decision-making process through recognition of the sustainability value of upcycled foods (53). The indirect effect of product knowledge through attitude indicates that knowledge not only reduces cognitive uncertainty but also promotes purchase intention by improving consumers’ overall evaluation of the product category (74).

## 6 Implications and limitations

### 6.1 Theoretical contributions

This study contributes to the literature by providing a contextualized explanation of consumer acceptance of upcycled foods. Existing research on sustainable food consumption has mainly examined relatively established product categories, such as organic foods, green foods, plant-based foods, and functional foods. Upcycled foods, however, combine food resource reuse, product innovation, and consumer cognitive uncertainty, making their acceptance process more contextually complex. By focusing on upcycled foods, this study shows how health consciousness, environmental awareness, and product knowledge shape consumers’ attitudes toward this food category, and how attitude, subjective norm, and perceived behavioral control further influence purchase intention. In doing so, the study extends the explanatory scope of sustainable food consumption research to an emerging food category.

This study also enriches the application of the extended theory of planned behavior in the context of emerging sustainable food consumption. The classic theory of planned behavior emphasizes the predictive roles of attitude, subjective norm, and perceived behavioral control in behavioral intention, but it pays relatively limited attention to the antecedents of attitude. By incorporating health consciousness, environmental awareness, and product knowledge as antecedents of attitude, this study suggests that consumers’ attitudes toward upcycled foods are not shaped only by general consumption preferences, but are closely related to their health concerns, environmental value orientation, and product understanding. This model specification moves the theory of planned behavior beyond explaining how attitude influences intention and toward explaining how attitude is formed, thereby strengthening its explanatory power for consumer acceptance of upcycled foods.

The study further identifies the mediating role of attitude in the relationships between health consciousness, environmental awareness, product knowledge, and purchase intention. Within the theoretical model proposed in this study, health consciousness, environmental awareness, and product knowledge are indirectly associated with purchase intention through attitude. This finding indicates that consumers’ concern for healthy eating, attention to resource waste and environmental consequences, and knowledge of upcycled foods can enter the purchase intention formation process through favorable evaluations of this food category. Accordingly, this study provides a psychological mechanism framework centered on “value cognition and product understanding—attitudinal evaluation—purchase intention” for explaining consumer acceptance of upcycled foods.

### 6.2 Practical implications

The findings suggest that promoting upcycled foods requires more than emphasizing their environmental value. Although food waste reduction and resource circularity are central to the meaning of upcycled foods, consumers are unlikely to accept such products if they remain uncertain about ingredient sources, nutritional value, processing safety, or product quality. Therefore, firms should communicate upcycled foods not only as sustainable products, but also as legitimate food products. Information about ingredient origin, nutritional composition, processing methods, quality control, and safety assurance should be presented in a clear and accessible way. Such communication can help consumers understand that upcycled foods are not simply low-value reprocessed products, but food innovations based on the regulated use of resources that still retain edible value or nutritional potential.

Consumer education is particularly important because product knowledge showed the strongest effect on attitude. This finding implies that the market acceptance of upcycled foods depends heavily on whether consumers can correctly understand what “upcycled” means. If the concept is left vague, consumers may associate upcycled foods with leftovers, near-expiry products, or inferior-quality processing. Firms, retailers, industry associations, and public agencies should therefore reduce conceptual ambiguity through consistent terminology, simple product explanations, visible labeling, and educational content. Packaging, e-commerce product pages, retail displays, short videos, and public information campaigns can all be used to explain where upcycled ingredients come from, how they are processed, and why their use contributes to food waste reduction. In this sense, consumer education should not be treated as an additional promotional activity, but as a basic condition for building category legitimacy.

The results also indicate that sustainability communication needs to be more concrete and verifiable. Consumers with higher environmental awareness are more likely to develop favorable attitudes toward upcycled foods, but environmental concern alone may not be sufficient if product claims are broad or difficult to evaluate. Instead of relying on general expressions such as “green,” “eco-friendly,” or “sustainable,” firms should explain the specific resource-use logic behind the product. For example, communication can clarify what type of food resource is being reused, why it would otherwise be underutilized, and how the product contributes to reducing food waste or improving resource efficiency. More transparent sustainability information can reduce consumer skepticism and help consumers connect personal food choices with broader food system sustainability.

The findings further suggest that public policy can play an important role in shaping consumer trust and market order. Because upcycled foods are still an emerging category, unclear labels or inconsistent market claims may easily increase consumer confusion. Policymakers and industry regulators can support this market by developing clearer standards for labeling, certification, ingredient disclosure, safety assurance, and environmental claims. Such standards would help consumers distinguish upcycled foods from leftovers, ordinary recycled products, or near-expiry foods, while also preventing exaggerated or ambiguous sustainability claims. Public agencies may also incorporate upcycled foods into food waste reduction education, sustainable diet campaigns, and community-based food literacy programs. These efforts could strengthen consumer understanding while linking individual purchase behavior with food policy goals.

Finally, the results highlight the importance of addressing multiple determinants of purchase intention simultaneously. The significant effects of attitude, subjective norm, and perceived behavioral control indicate that consumers’ intentions to purchase upcycled foods are shaped by both individual evaluations and contextual influences. The significant effects of subjective norm and perceived behavioral control suggest that consumers are more likely to consider upcycled foods when they perceive social approval and believe that purchasing them is convenient. Firms can strengthen social recognition through expert endorsement, credible consumer reviews, and cooperation with nutrition professionals, food safety experts, and sustainability organizations. At the same time, product availability, price acceptability, channel visibility, and information transparency should be improved. These efforts are particularly important because consumers’ purchase intentions are influenced not only by their attitudes toward upcycled foods but also by their perceptions of social expectations and the ease of purchasing such products. Market development should therefore combine consumer education with practical access, allowing consumers not only to understand upcycled foods, but also to choose them easily in everyday purchasing situations.

### 6.3 Limitations and future research

This study has several limitations. The sample consisted of adult consumers in China, which provides empirical evidence for understanding consumer acceptance of upcycled foods in the Chinese market. However, consumers in different countries and regions may differ in their perceptions of food waste, trust in food safety, sustainable consumption culture, income levels, and the maturity of the upcycled food market. Therefore, the generalizability of the findings to other cultural and market contexts requires further examination. Future research could conduct cross-national or cross-cultural comparisons to explore whether consumers’ attitudes and purchase intention toward upcycled foods vary across institutional environments, consumption cultures, and food policy contexts.

The model in this study focused on health consciousness, environmental awareness, product knowledge, attitude, subjective norm, and perceived behavioral control, which together explain important psychological pathways underlying consumers’ purchase intention toward upcycled foods. Nevertheless, consumer decision-making in this context may also be influenced by other factors. For example, perceived food safety, perceived risk, trust, perceived naturalness, price sensitivity, taste expectations, and brand familiarity may all shape consumers’ evaluations and choices. Future studies could incorporate risk- and trust-related mechanisms while maintaining theoretical clarity, thereby offering a more nuanced understanding of the dual psychological process in which consumers may recognize the sustainability value of upcycled foods while still holding concerns about product quality.

This study did not further distinguish among different types of upcycled foods. In practice, upcycled foods may include bakery products, beverages, snacks, cereal products, condiments, functional foods, and other categories. These products may differ in processing methods, health associations, price acceptability, taste expectations, and consumer familiarity. For instance, consumers may respond differently to beverages made from fruit and vegetable by-products, bakery products made from cereal by-products, or upcycled functional foods with nutritional enhancement claims. Future research could compare how product categories, labeling information, nutrition claims, and certification cues influence consumer acceptance, thereby providing a more fine-grained understanding of contextual differences in upcycled food consumption.

## Data Availability

The original contributions presented in the study are included in the article/Supplementary material, further inquiries can be directed to the corresponding authors.
