# Supplementary material for: Understanding purchase intention toward upcycled foods through a consumer education lens: an extended theory of planned behavior approach
Source: Front Nutr. 2026 Jul 24;13:1871592. doi: 10.3389/fnut.2026.1871592 (PMC13447081; doi:10.3389/fnut.2026.1871592)
Supplement: Supplementary file 1 [file Table_1.DOCX]

Appendix A.

Table A1. Measurement Items

| Construct | Code | Item |
| --- | --- | --- |
| Health Consciousness | HC1 | I am very concerned about the impact of my daily diet on my health. |
|  | HC2 | When purchasing food, I usually pay attention to whether it is beneficial to health. |
|  | HC3 | I tend to choose foods that help maintain or improve my health. |
|  | HC4 | I pay close attention to the nutritional value and health-related attributes of foods. |
| Environmental Awareness | EA1 | I believe that individual food consumption behavior has an impact on the environment. |
|  | EA2 | I pay attention to resource waste in food production and consumption. |
|  | EA3 | I am concerned about food waste and its environmental consequences. |
|  | EA4 | When purchasing food, I consider the environmental impact of the product. |
| Product Knowledge | PK1 | I understand the basic meaning of upcycled foods. |
|  | PK2 | I know that upcycled foods are often made from food resources that still have edible or nutritional value. |
|  | PK3 | I understand the role of upcycled foods in reducing food waste and improving resource use efficiency. |
|  | PK4 | I can distinguish upcycled foods from leftover foods or low-quality reprocessed foods. |
| Attitude | ATT1 | I think purchasing upcycled foods is a good choice. |
|  | ATT2 | I have a positive attitude toward purchasing upcycled foods. |
|  | ATT3 | I believe that purchasing upcycled foods is valuable. |
|  | ATT4 | Overall, I approve of purchasing upcycled foods. |
| Subjective Norm | SN1 | People who are important to me would support me in purchasing upcycled foods. |
|  | SN2 | My family or friends would approve of my purchasing upcycled foods. |
|  | SN3 | People around me have positive views about purchasing upcycled foods. |
|  | SN4 | Most people whose opinions I value would approve of my purchasing upcycled foods. |
| Perceived Behavioral Control | PBC1 | If I want to purchase upcycled foods, I believe I can do so. |
|  | PBC2 | For me, purchasing upcycled foods would be relatively easy. |
|  | PBC3 | I can obtain enough information to decide whether to purchase upcycled foods. |
|  | PBC4 | I have the resources and ability to purchase upcycled foods if they are available. |
| Purchase Intention | PI1 | I am willing to purchase upcycled foods in the future. |
|  | PI2 | If I have the opportunity, I would consider purchasing upcycled foods. |
|  | PI3 | I am willing to try upcycled foods when they are available. |
|  | PI4 | I am willing to recommend upcycled foods to others. |
